# Incomplete recording of Indigenous identification status under-estimates the prevalence of Indigenous population attending Australian general practices: a cross sectional study

**DOI:** 10.1186/s12913-019-4393-6

**Published:** 2019-08-14

**Authors:** Belinda K. Ford, Marlene Kong, James S. Ward, Jane S. Hocking, Christopher K. Fairley, Basil Donovan, Rebecca Lorch, Simone Spark, Matthew Law, John Kaldor, Rebecca Guy

**Affiliations:** 1 Kirby Institute, Faculty of Medicine, UNSW Sydney, Sydney, NSW Australia; 2 South Australian Health and Medical Research Institute, Adelaide, SA Australia; 3 Centre for Epidemiology and Biostatistics, Melbourne School of Population and Global Health, University of Melbourne, Melbourne, Victoria Australia; 4 Monash University Central Clinical School and Melbourne Sexual Health Centre, Melbourne, Victoria Australia; 5 School of Public Health and Preventive Medicine, Monash University, Melbourne, Victoria Australia

**Keywords:** Aboriginal and/or Torres Strait islander people, Indigenous status, Medical records, Health data, General practice

## Abstract

**Background:**

Australian Aboriginal and Torres Strait Islander (Indigenous) peoples face major health disadvantage across many conditions. Recording of patients’ Indigenous status in general practice records supports equitable delivery of effective clinical services. National policy and accreditation standards mandate recording of Indigenous status in patient records, however for a large proportion of general practice patient records it remains incomplete. We assessed the completeness of Indigenous status in general practice patient records, and compared the patient self-reported Indigenous status to general practice medical records.

**Methods:**

A cross sectional analysis of Indigenous status recorded at 95 Australian general practices, participating in the Australian Chlamydia Control Effectiveness Pilot (ACCEPt) in 2011. Demographic data were collected from medical records and patient surveys from 16 to 29 year old patients at general practices, and population composition from the 2011 Australian census. General practitioners (GPs) at the same practices were also surveyed. Completeness of Indigenous status in general practice patient records was measured with a 75% benchmark used in accreditation standards. Indigenous population composition from a patient self-reported survey was compared to Indigenous population composition in general practice records, and Australian census data.

**Results:**

Indigenous status was complete in 56% (median 60%, IQR 7–81%) of general practice records for 109,970 patients aged 16–29 years, and Indigenous status was complete for 92.5% of the 3355 patients aged 16–29 years who completed the survey at the same clinics. The median proportion per clinic of patients identified as Indigenous was 0.9%, lower than the 1.8% from the patient surveys and the 1.7% in clinic postcodes (ABS). Correlations between the proportion of Indigenous people self-reporting in the patient survey (5.2%) compared to status recorded in all patient records (2.1%) showed a fair association (r = 0.6468; *p* < 0.01). After excluding unknown /missing data, correlations weakened.

**Conclusions:**

Incomplete Indigenous status records may under-estimate the true proportion of Indigenous people attending clinics but have higher association with self-reported status than estimates which exclude missing/unknown data. The reasons for incomplete Indigenous status recording in general practice should be explored so efforts to improve recording can be targeted and strengthened.

**Trial registration:**

ACTRN12610000297022. Registered 13th April 2010.

## Background

Australian Aboriginal and Torres Strait Islander (hereafter Indigenous) peoples face major health disadvantage across a wide range of health conditions; including cancer, diabetes, and sexually transmissible infections (STIs) [1–3]. Reducing the persisting disparity of Indigenous peoples’ health is a key commitment of national health strategies and the Closing the Gap (CTG) framework [1, 4].

In Australia, more than half of the Indigenous population access healthcare through mainstream primary care services [5]. Recording of a patients’ Indigenous status in practice records is important to ensure optimal health care and evaluate the effectiveness of clinical practices services and programs [6] recommended for Indigenous patients. For example, Indigenous adults are recommended to have annual health checks (Medicare Benefits Schedule (MBS) items 715 or 10,987) which provide preventive health strategies such as STI screening and detection of chronic disease risk factors [7]; and the CTG Pharmaceutical benefits scheme (PBS) co-payment reduces out-of-pocket costs of medicines for Indigenous people [8]. Additionally, for health services, recognition of patients’ as Indigenous is required for health services to claim incentive payments, such as Medicare Australia’s Practice Incentive Program (PIP) Indigenous Health Incentives [9], which can support delivery of quality services. Finally, complete Indigenous status in patient files provides a more accurate picture for monitoring disease prevalence, to identify risk and plan effective services and programs [6].

**Fig. 1:**
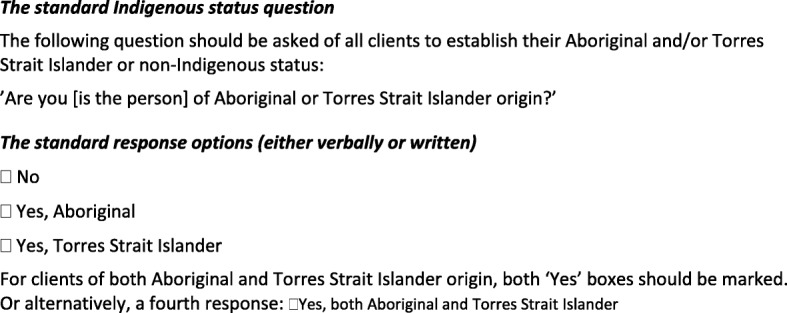
National Best Practice guidelines for collecting Indigenous status in health data sets [6]

National guidelines for the collection of Indigenous identification status in health data sets recommend the use of a standard verbal or written question (Fig. 1); and that service providers make completion of Indigenous status a mandatory requirement for new patient registrations [6]. The Royal Australian College of General Practitioners (RACGP) [10, 11] recommends at least 75% of patient records be complete for cultural background where clinically relevant to achieve practice accreditation, including Indigenous status recording [10]. A number of general practice audits have shown that a large proportion of patient records did not have an Indigenous status completed [12–15]. However to our knowledge no studies have compared the estimates of Indigenous population composition in general practice with other data sources, to provide an indication of how incomplete recording may under-estimate the true prevalence of the Indigenous population attending the clinics.

This study was completed in the context of the Australian Chlamydia Control Effectiveness Pilot (ACCEPt), a randomised controlled trial to determine if annual chlamydia testing for 16 to 29 year olds in general practice can reduce chlamydia prevalence. We took the opportunity to assess the completeness of Indigenous identification status recording (Aboriginal, Torres Strait Islander, both Aboriginal and Torres Strait Islander or neither) in general practice clinics, and examined factors associated with clinicians having greater than 75% completion in patient records. We also used a reference of patient self-reported Indigenous status to measure agreement of Indigenous status recording across administrative data sources, including complete and incomplete general practice medical records.

## Methods

### Setting

The ACCEPt trial was based in 52 towns in Victoria, New South Wales (NSW), Queensland (QLD) and South Australia (SA). All 152 primary care services (general practice and Aboriginal Community Controlled Health Services [ACCHSs]) located within these towns participated in ACCEPt; 134 were in regional and remote areas and nine in a major metropolitan city. The 2011 Australian Census reported that a higher proportion of Indigenous people reside in inner regional (22%) and outer regional /remote (30%) areas than in major cities (35%). NSW has the highest Indigenous population, around one-third (31%) of Indigenous people reside there, followed closely by QLD (28%) [16].

The study population used in these analyses was restricted to 44 of the 52 towns (95 clinics) with general practice clinics only, and which had data available for all data sources. We excluded all clinics located in towns (8 clinics) with an ACCHSs due to the known high completeness of Indigenous identification status recording in ACCHSs [14].

### Study design

This cross-sectional study assessed completeness and agreement of Indigenous status recording in general practice using four data sources (described below); three from the ACCEPt trial and included i) general practice electronic patient records, ii) a patient survey, iii) a general practitioner survey [17] and the fourth was Australian Bureau of Statistics (ABS) population census data. Since data sources were independent and de-identified, patient self-reported Indigenous status was used to assess the agreement of Indigenous status recording compared to general practice and population data.

### Data sources

#### Patient medical records

Using strict confidentiality and privacy processes, de-identified information of all 16–29 year olds who attended 95 general practice clinics participating in ACCEPt during 2011 was extracted from patient information management systems using software called GRHANITE™ (‘GeneRic Health Network Information Technology for the Enterprise’) [18]. Variables collated included routinely collected patient ID, clinic number, clinician identifier, gender, age, Indigenous identification status, and attendance date.

#### Patient survey

Between 2010 and 2012, a cross-sectional behavioural and biological patient survey was conducted to determine risk factors and the chlamydia prevalence in each town as they commenced ACCEPt. At each clinic, sexually active men and women aged 16–29 years were recruited consecutively to the survey when they attended for a GP consultation. The survey has been described in detail elsewhere [19]. In brief, participants completed a self-reported questionnaire on a hand-held computer, including demographics (age, gender, Indigenous identification status), sexual behaviours, chlamydia knowledge, and clinic attendance information. Overall 4284 participants participated, with a response rate of 65.7% [19]. These analyses use patient survey data from the 95 clinics described above (*n* = 3355). Completeness of responses to the Indigenous status question was 92.5%.

#### Clinician survey

A cross sectional survey of GPs participating in ACCEPt was conducted during study recruitment meetings. GPs unable to complete the questionnaire at that time were given a reply-paid envelope to return the survey, with follow up reminders for non-responders. The survey details have been published elsewhere [20, 21]. In brief, self-administered, paper questionnaires captured clinicians’ demographics (gender, age), experience and training (country of primary medical degree and number of years worked in general practice), chlamydia knowledge, awareness and testing practices, including whether GPs routinely collected patients’ Indigenous identification status, and what proportion of patients the GPs thought were Indigenous. Of the 773 GPs enrolled in ACCEPt, there was a response rate of 72% overall [21]. Responses from 496 (64%) GPs based at the 95 clinics described above were included in these analyses.

#### Australian Bureau of Statistics (ABS census)

ABS 2011 population census usual resident count data [22] were used to calculate the proportion of 16–29 year old Indigenous people for each of the 44 postcodes (95 clinics); and to determine the remoteness of clinics using the Australian Statistical Geographical standard (ASGS) classifications of major city, inner regional, outer regional, remote and very remote [22].

### Analysis

#### Completeness of Indigenous identification status

To assess the completeness of Indigenous identification status in the electronic patient records, the proportion of patients aged 16–29 years who had an Indigenous identification status recorded was calculated. We adopted a benchmark of 75% completeness of Indigenous status as the RACGP recommends that greater than 75% of patient records be complete for cultural background [10] and we stratified clinics as performing above or below.

To assess GP factors associated with high completion of Indigenous identification status (> 75% completion) in patient records, we used a univariate and multivariate logistic regression. Variables included in the model were remoteness, jurisdiction, GPs’ responses to routinely asking about patients’ Indigenous identification status; and GPs perceived proportion of Indigenous patients attending their clinic. Factors with a *p* < 0.05 in the univariate analysis were included in the multi-variate analysis.

#### Population composition of Indigenous people recorded in different data sources

We estimated the proportion of populations aged 16–29 years that identified as Indigenous for each data source: patient self-report, general practice patient records, and ABS postcodes. We calculated the overall proportion using the total number of Indigenous patients aged 16–29 years recorded across all clinics, as well as the median proportion and the interquartile range across all clinics. We focused on the median, due to the large variability in size of the clinics and the potential for larger clinics to influence the overall sample results.

For general practice records we used two different denominators; i) all patient records (includes those with a missing or unknown Indigenous identification status) and ii) patient records where those with missing or unknown Indigenous identification status are excluded (complete case analysis).

#### Agreement of Indigenous status recording across data sources

Since all data sources were de-identified it was not possible to use data linkage to assess accuracy of recording in general practice records. Patient self-report is the recommended approach for collecting Indigenous status in health data sets [6]. Therefore the patient survey self-reported Indigenous status was used to measure the agreement of Indigenous status recording in the general practice and ABS data sources. We used a Pearson’s correlation to explain the direction and strength of the associations between self-reported Indigenous status and that found in the patient medical records (all patient records) and ABS census. We repeated the above correlations using only complete case patient records.

## Results

The study population included 95 general practice clinics located in NSW (28.4%), QLD (26.3%), Victoria (41.1%) and SA (4.2%). Clinics were located in major cities (9.7%), inner regional (72.6%), and outer regional or remote towns (17.7%). From the patient information management systems, we extracted records for 109,970 (clinic range: 60–12,120) patients aged 16–29 years old attending clinics in 2011.

### Completeness of Indigenous identification status in patient records

Indigenous identification status was found to be complete for 61,086 (55.5% of all patient records aged 16–29 year olds (median of 60% per clinic) (Table 1). One-third of clinics (35%) had greater than 75% completion of Indigenous identification status in patient records.

**Table 1:** Completeness of Indigenous status in general practice medical records

	Medical records of patients aged 16–29 year olds attending 95 general practices
Total records	109,970
Overall proportion of combined clinic records with a complete Indigenous status (n, %)	61,086 (55.5%)
Median proportion per clinic of records with a complete Indigeous status (%, IQR)	60%, (IQR 7–81%)

In the c*linician survey*, 496 GPs from the 95 clinics provided a response; and 132 (27%) GPs were found to have greater than 75% complete recording of Indigenous status in their clinic patient records. GP characteristics and recording practices are listed in Table 2. A small proportion (5.6%) of GPs reported they always asked patients about their Indigenous identification status, 52.2% sometimes asked and 32.7% never asked. Most GPs (61.3%) thought that less than 1% of patients attending their clinic were Indigenous, with a further 17.9% reporting they were “unsure” of their clinics’ Indigenous attendees.

**Table 2:** General practitioner characteristics associated with > 75% completeness of Indigenous identification status in the patient records

General practitioner characteristics (Clinician survey)	GPs survey responses (%)		Factors associated with GPs having > 75% complete recording of Indigenous identification status in patient records* (*n* = 496)	
	All GPs (*n* = 496)	GPs with > 75% complete recording in patient records (*n* = 132)	OR, (95% CI, *p* value)	AOR, (95% CI, *p* value)
Gender				
Male	60.1	57.5	1	
Female	39.9	42.4	1.15 (0.77 to 1.73, *p* = 0.49)	
Age (years)				
< 44 years	47.8	52.3	1	
> 45 years	52.2	47.7	0.78 (0.53 to 1.17, *p* = 0.23)	
Country obtained primary medical degree				
Australia	57.7	55.3	1	
Outside Australia	42.3	44.7	1.14 (0.76 to 1.70, *p* = 0.52)	
Years working in general practice (*n* = 492)				
≤ 10 years	47.8	48.9	1	
> 10 years	52.2	51.1	0.94 (0.63 to 1.41, *p* = 0.77)	
Perception of the proportion of Indigenous patients that attend their clinic (*n* = 496)				
< 1%	60.7	52.3	1	1
2–5%	17.1	22.7	**1.81 (1.07 to 3.06,** ***p*** **= 0.03)**	1.28 (0.69 to 2.40, *p* = 0.44)
> 5%	3.2	7.0	**4.49 (1.61 to 12.51, p = < 0.01)**	2.59 (0.76 to 8.87, *p* = 0.13)
Not sure	17.9	18.0	1.22 (0.70 to 2.10, *p* = 0.48)	1.13 (0.63 to 2.07, *p* = 0.67)
Self- reported routinely ask Indigenous identification status of patients				
Never	32.7	26.7	1	1
Sometimes	52.2	49.6	1.22 (0.76 to 1.94, *p* = 0.41)	1.23 (0.73 to 2.10, *p* = 0.43)
Usually	8.9	11.5	1.88 (0.91 to 3.88, *p* = 0.09)	1.31 (0.55 to 3.08, *p* = 0.54)
Always	5.6	12.2	**4.84 (2.10 to 11.17, p < 0.01)**	**4.08 (1.59 to 10.47, p < 0.01)**
Remoteness location				
Major City	9.7	18.1	1	1
Inner Regional	72.6	62.1	**0.29 (0.16 to 0.55, p < 0.01)**	**0.11 (0.56 to 0.24,** ***p*** **< 0.01)**
Outer Regional/ Remote	17.7 (Remote = 2.0)	19.7	**0.42 (0.20 to 0.87,** ***p*** **= 0.02)**	**0.09 (0.03 to 0.22, p < 0.01)**
State				
NSW	17.7	24.2	1	1
QLD	22.8	37.8	1.39 (0.78 to 2.46, *p* = 0.26)	1.57 (0.84 to 2.92, *p* = 0.155)
Victoria/SA	59.5 (SA = 4.8)	37.8	**0.36 (0.21 to 0.61,** ***p*** **< 0.01)**	**0.28 (0.14 to 0.55,** ***p*** **< 0.01)**

Boldface values were significant at *p*<0.05

In the univariate logistic regression (Table 2), GP characteristics that were associated with > 75% completion of Indigenous identification status in 16–29 year old patient records were: GPs who reported *always* asking clients their Indigenous identification status’, and GPs’ perception that greater than 5%, or between 2 and 5% of clinic attendees were Indigenous. GP characteristics that were less likely to be associated with > 75% completion of Indigenous identification status were: clinics located in inner regional, and outer regional / remote areas; or those located in the States of Victoria or South Australia. These factors all remained significant in the multivariate model, except for GPs perceptions of the proportion of Indigenous clinic attendees (Table 2).

### Population composition of Indigenous people recorded in different data sources

The population composition of Indigenous and non-Indigenous people for each data set is provided in Table 3.

**Table 3:** Indigenous population composition reported in each data source for people aged 16–29 year olds

Data source	Recorded as Indigenous		Recorded as non-Indigenous	
	*Proportion in combined data (%)*	*Median proportion per clinic (%, IQR)*	*Proportion in combined data (%)*	*Median proportion per clinic (%, IQR)*
*Patient survey*	5.2	1.8 (0–7.3)	87.3	92.9 (82.0–97.8)
*All patient records*	2.1	0.9 (0.2–3.1)	53.4	70.4 (43.8–90.2)
Patient records (complete case)*	3.7	3.4 (1.2–15.4)	96.3	96.5 (84.6–98.8)
*ABS Census 2011*	1.6	1.7 (1.0–4.3)	92.1	93.6 (90.2–95.0)

*complete case analysis used 44.5% of all records (n = 48,884) where records listed with a *missing/ unknown* Indigenous status were excluded

#### Patient medical records

Overall, 2.1% of 16 to 29 year olds were recorded as Indigenous in patient medical records and the median proportion of 16–29 year olds recorded as Indigenous per clinic was 0.9%. The median proportion per clinic of non-Indigenous patients was 70.4 and 53.4% of combined clinic records. Using a complete case analysis of Indigenous status in patient medical records (44.5% *n* = 48,884), Indigenous people represented a higher median proportion per clinic of 3.4% among the 16–29 year old patient population, and 3.7% of combined clinic records.

#### Patient survey

In the *patient survey*, 3355 patients aged 16–29 years responded to the chlamydia prevalence survey, there was a 92.5% completeness of responses to the Indigenous status question. The clinic median proportion of patient identifying as Indigenous was 1.8%, and 173 (5.2%) patients overall.

#### ABS population

The *2011 ABS census* estimated a median proportion per postcode of Indigenous people aged 16–29 years in the 44 clinic postcodes as 1.7%, and overall was 1445 (1.6%) people.

### Agreement of Indigenous status recording across data sources

Correlations between the proportion of Indigenous people self-reporting in the patient survey (5.2%) and all patient records (2.1%) showed a fair association (r = 0.6468; *p* < 0.01) (Table 4). After excluding records with an unknown and missing Indigenous status (complete case analysis), the correlation between weakened (r = 0.3136; *p* = 0.002) (Table 4).

**Table 4:** The proportion of Aboriginal and Torres Strait Islander (Indigenous) people aged 16–29 years recorded in patient records, 2011 ABS Census, and patient survey; and correlations between data sources

Data source	Correlations between data sources			
	*Patient survey*	*All patient records*	*Patient records (complete case)*	*ABS Census 2011*
*Patient survey*	1.00	0.6468 (*p* < 0.01)	0.3136 (*p* < 0.01)	0.7010 (*p* < 0.01)
*All patient records*	–	1.00	0.1361 (*p* = 0.19)	0.7005 (*p* < 0.01)
Patient records (complete case)*	–	–	1.00	0.1948 (*p* = 0.06)
*ABS Census 2011*	–	–	–	1.00

*Complete case analysis- 44.5% of records (*n* = 48,884) where records listed with a *missing/ unknown* Indigenous status were excluded

The proportion of Indigenous people self-reporting in the patient survey was also correlated with ABS data (1.6%) showing a moderate association (r = 0.7010; p < 0.01) (Table 4).

## Conclusions

This study found that almost half (44%) of patient records in the 95 general practice clinics had unknown or missing Indigenous identification status recorded. Using all patient records, the median proportion per clinic of patients identified as Indigenous was 0.9%, much lower than the 1.8% from the patient survey at the same clinics and the 1.7% in the clinic postcodes according to the ABS census data. The gaps in recording of Indigenous identification status are concerning but consistent with other studies [3, 14, 15]. However, to our knowledge this is the first study that assesses how incompleteness may bias the actual prevalence of Indigenous population attending general practice clinics through comparison with patient self-reported Indigenous status and population data.

We found that only 5.6% of GPs surveyed always asked patients about their Indigenous identification status, and these GPs were over four times more likely to have complete patient records. Additionally, GPs from regional and remote areas had poorer completeness, despite larger proportions of Indigenous populations residing in these areas [22]. This finding differs to a previous study which showed regional and remote areas to have more complete records [13], and may have been influenced by better recording at the major city clinics included in our study. Health policy and GP training initiatives for Indigenous status recording in general practice should be strengthened in an effort to improve completeness, targeting the areas with known poorer recording. However, the success of such initiatives would require a thorough understanding of the barriers and facilitators to recording from the perspectives of both clinic staff and Indigenous people, including staff cultural awareness [23] and application in existing clinic systems [15, 23], appropriate ways to ask and explain why Indigenous status is being recorded [15] and willingness of patients to disclose due to fear of racial discrimination, social or historical factors [24].

In addition to delivering culturally safe care, Indigenous identification status is useful for disease monitoring and surveillance. Our study also showed that when complete case analysis was used for general practice records, there were poorer correlations with patient self-report than when all patient records were used; and the estimated median proportion of Indigenous people increased from 0.9% in all records to 3.4% in the complete case analysis. Therefore, population disease estimates relying on general practice data, which exclude the records with missing or unknown Indigenous status, are likely to bias and possibly over-estimate rates among Indigenous people. A Western Australian study using data linkage between an infectious disease surveillance database and established health administrative databases to improve accuracy of Indigenous identification status, found that rates of chlamydia and syphilis among Indigenous people had been overestimated by up to 30%, compared to the pre-linked rates which excluded cases of unknown Indigenous status [25].

There are a few potential limitations to consider in our analysis. First, the study was conducted in selected general practices and towns in four Australian States and may not be representative of clinics across Australia, particularly those in major cities. However, it remains important since Indigenous populations reside in these regional and remote areas [16] and across health care indices rates are higher for Indigenous populations in regional areas [2]. Second, there is a reported undercount of Indigenous status for both Indigenous and non-Indigenous people in the 2011 census [22]; and further the ABS data focused on the entire postcode, and was compared to records for only patients who had attended the general practice that year, some of whom may have travelled from other postcodes. A study by Kong et al. showed that 74.8% of 16–29 year old Australians, attend a general practice at least once each year [26]. However annual health care attendance would have only influenced our study if variations in attendance between Indigenous and non-Indigenous people exist.

In conclusion, around half the patient records in this study had missing or unknown Indigenous identification status, highlighting that further efforts are needed to improve completeness of Indigenous identification status in general practices. Complete and accurate recording of Indigenous identification status in patient records may allow health care providers to better address the healthcare needs of Indigenous people; and may enable better health policy decisions addressing the needs of Indigenous people.

## Data Availability

The datasets used and/or analysed during the study are available from the corresponding author on reasonable request.

## Abbreviations

ABS: Australian Bureau of Statistics
ACCEPt: Australian Chlamydia Control Effectiveness Pilot
ACCHSs: Aboriginal Community Controlled Health Services
CTG: Closing the Gap
GPs: General practitioners
GRHANITE™: GeneRic Health Network Information Technology for the Enterprise
MBS: Medicare Benefits Schedule
NSW: New South Wales
PBS: Pharmaceutical benefits scheme
PIP: Practice Incentive Program
QLD: Queensland
RACGP: Royal Australian College of General Practitioners
SA: South Australia
STIs: Sexually transmissible infections
